# Editorial: The role of the brain in health and disease across the lifespan

**DOI:** 10.3389/fnhum.2023.1272772

**Published:** 2023-09-05

**Authors:** Shannon D. Donofry, Chelsea M. Stillman, Irene Esteban-Cornejo

**Affiliations:** ^1^RAND Corporation, Pittsburgh, PA, United States; ^2^Department of Psychology, University of Pittsburgh, Pittsburgh, PA, United States; ^3^Department of Physical Education and Sports, Faculty of Sport Sciences, Sport and Health University Research Institute (iMUDS), University of Granada, Granada, Spain; ^4^Instituto de Investigación Biosanitaria ibs.GRANADA, Granada, Spain; ^5^Centro de Investigación Biomédica en Red Fisiopatología de la Obesidad y Nutrición (CIBERobn), Instituto de Salud Carlos III, Madrid, Spain

**Keywords:** brain health, cognitive health, health neuroscience, health psychology, chronic disease risk

Advances in the fields of human neuroscience and health psychology have reaffirmed that there is a dynamic reciprocal relationship between the brain and the body, and that the health of one system directly influences the health of the other. This observation has prompted the emergence of the field of health neuroscience, which leverages the conceptual frameworks and methodologies of multiple disciplines (e.g., health psychology, cognitive neuroscience, and exercise neuroscience) to better understand how the brain affects and is affected by physical health broadly defined (Erickson et al., 2014). In so doing, the goal is to uncover new mechanistic insights about the role of the brain in health and chronic disease risk to inform the development of more effective intervention and prevention initiatives, influence health policies, and promote greater quality of life across the lifespan. In support of these efforts, this Research Topic brought together scholars from complementary fields such as clinical and health psychology, cognitive psychology, public health, and neuroscience to build a Research Topic of articles centered on the multifaceted role of the brain in health and disease across the lifespan.

Two of the included articles explored the impact of genetic polymorphisms implicated in risk for cardiovascular and neurodegenerative diseases on brain function in midlife adults with overweight and obesity. The goal of these projects was to explore whether individuals with high-risk genetic variants exhibit pre-clinical differences in brain function that may be indicative of accelerated neurocognitive aging. The first article describes an investigation of the relationship between carrying the apolipoprotein E ε4 allele, overweight/obesity status, and brain activity during a working memory task among middle-aged adults (Drake et al.). The results indicated that middle-aged individuals with overweight/obesity and the ε4 allele exhibited reduced brain activity in regions associated with working memory. The second article explored the impact of Inter-individual variation in the fat mass and obesity-associated (FTO) gene on resting cerebral blood flow (rCBF) among midlife individuals with overweight and obesity enrolled in a randomized controlled trial of a behavioral weight loss intervention (Stillman et al.). The FTO gene has long been linked to body weight, and higher body weight is known to relate to poorer brain health. The investigators found that individual differences in rCBF may be attributable to a common FTO variant. However, the weight loss intervention was equally effective at increasing rCBF regardless of FTO genotype, suggesting that the effects of overweight and obesity on rCBF are responsive to lifestyle change and, therefore, potentially reversible. Together, these findings indicate that interventions targeting modifiable risk factors, such as lifestyle modifications, may be important for mitigating cognitive decline in individuals at higher genetic risk.

Two other articles in this Research Topic explored the relationship between functional and structural connectivity within key brain networks and health-related outcomes. The first study aimed to examine the association of body mass index (BMI) with seed-based resting state functional connectivity (rsFC) of the hippocampus and amygdala among 34 women with breast cancer (Donofry et al.). A secondary aim was to assess whether any BMI-related differences in rsFC were related to psychological health outcomes in this patient group. Higher BMI was associated with weaker rsFC between hippocampal and amygdala seeds and several prefrontal brain regions supporting executive control. Further, weaker rsFC between the hippocampus and prefrontal cortex was associated with more anxiety symptoms in the women, suggesting that the strength of certain brain pathways relate to both physical and psychological health outcomes in a patient population at high risk for psychological distress. The second study adopted a longitudinal approach to explore how poor health behaviors, in this case cannabis use, impacts the development of white matter pathways in adolescents (Lichenstein et al.). Specifically, this study investigated the relationship between cannabis use during adolescence and emerging adulthood and the microstructure of the cingulum and anterior thalamic region (ATR). Moderate cannabis use during adolescence was associated with increased fractional anisotropy (FA) in the cingulum and ATR at age 20, compared to minimal and heavy use. However, moderate and heavy cannabis use from ages 12–21 was linked to reduced positive change in FA in the cingulum between ages 20 and 22, relative to minimal use. These longitudinal findings suggest that cannabis exposure may delay the maturation of the cingulum during the transition to adulthood, potentially impacting individuals' functioning in later stages of development.

Contributors to this Research Topic also used electroencephalogram (EEG) to evaluate the impact of chronic disease on brain function. The first of these studies explored the impact of continuous positive airway pressure (CPAP) treatment for sleep apnea on EEG-measured components of sleep architecture known to be detrimentally impacted by sleep apnea (Wilckens et al.). In a study involving adults with comorbid type 2 diabetes, spectral analysis of EEG during sleep was performed to compare individuals treated with CPAP vs. sham CPAP. While there were no significant differences in spectral characteristics of sleep architecture between the two groups, the SHAM group transitioning to active CPAP treatment demonstrated increased sigma activity and decreased beta activity during sleep. CPAP treatment also resulted in a reduction in slow oscillation power during the initial non-rapid eye movement (NREM) period, particularly in the frontal EEG channels. These findings suggest that CPAP may enhance spindle activity, mitigate neurophysiological arousal, and improve sleep quality, highlighting the neurophysiological mechanisms, and potential benefits of CPAP treatment. The second study sought to identify novel brain-based biomarkers of Malnutrition-inflammation complex syndrome (MICS), common in End Stage Renal Disease (Jatupornpoonsub et al.). Existing assessments for MICS are invasive, time-consuming, and need to be repeated multiple times in order to detect MICS onset. The goal of this cross-sectional study was to investigate whether quantitative EEG could serve as a reliable alternative to existing tests for MICS. Patients with End Stage Renal Disease and health controls completed the traditional testing for MICS, as well as a resting EEG. Several EEG parameters were evaluated for their correlations with MICS symptoms. EEG parameters associated with the duration of certain microstates were used to form a MIC Index that reliably predicted MICS risk with 100% accuracy. This study demonstrates how EEG parameters can be useful biomarkers of disease states and hold promise for replacing inefficiencies in existing diagnostic approaches.

Finally, one study outlines the research protocol being employed to explore the benefits of resistance exercise on measures of neurocognitive aging among older adults (Solis-Urra et al.). Previous research has shown that exercise interventions, particularly those that employ aerobic exercise, have positive effects on cognitive functioning in older adults. However, it remains unclear whether resistance exercise yields similar benefits. The AGUEDA study proposes to address these gaps in the literature by randomizing 90 cognitively healthy older adults to either a 24-week resistance exercise program or a waitlist control group to examine the impact of the intervention on executive function, brain markers (including Aβ deposition, gray and white matter measures, functional connectivity, and cerebral blood flow), peripheral molecular markers, and other cognitive outcomes. The study also aims to identify potential mediators and moderators of the exercise-induced improvements. This study will address a major limitation of existing literature and has the potential to reveal novel mechanistic pathways through which exercise influences brain health in late adulthood.

The studies presented in this Research Topic contribute to a better comprehension of brain health and its impact on various health-related outcomes, and the implications of these findings are noteworthy. The identification of brain-based biomarkers for diseases like Malnutrition-Inflammation Complex Syndrome could lead to more efficient and accurate diagnostic methods. Understanding the impact of health behaviors, such as cannabis use, on the developing brain has implications for early interventions and targeted prevention strategies. Furthermore, the research underscores the importance of lifestyle modifications, such as weight management and exercise, in promoting brain health and mitigating cognitive decline. By focusing on modifiable risk factors, personalized interventions can be developed to improve outcomes for individuals determined to be at higher risk for cognitive decline. The work presented in this Research Topic exemplifies the power of interdisciplinary collaboration and the potential of state-of-the-science methodologies in advancing our understanding of the brain's role in health and disease. The knowledge gained through these efforts will help to facilitate a more comprehensive approach to healthcare, fostering wellbeing and resilience across the lifespan.

## Author contributions

SD: Conceptualization, Data curation, Formal analysis, Writing—original draft, Writing—review and editing. CS: Conceptualization, Writing—original draft, Writing—review and editing. IE-C: Writing—original draft, Writing—review and editing.
